# Reply to correspondence ‘Conserved signatures indicate HIV-1 transmission is under strong selection and thus is not a “stochastic” process’ by Gonzalez et al., Retrovirology 2017

**DOI:** 10.1186/s12977-017-0332-y

**Published:** 2017-02-24

**Authors:** Corinna S. Oberle, Carsten Magnus, Beda Joos, Peter Rusert, David Beauparlant, Roger Kouyos, Alexandra Trkola, Karin J. Metzner, Huldrych F. Günthard

**Affiliations:** 1 Division of Infectious Diseases and Hospital Epidemiology, University Hospital Zurich, University of Zurich, Zurich, Switzerland; 2 Institute of Medical Virology, University of Zurich, Zurich, Switzerland; 3 Department of Biosystems Science and Engineering, ETH Zürich, Basel, Switzerland

In a correspondence in this issue of Retrovirology [1], Gonzalez et al. comment on our study published in Retrovirology in September 2016 [2] where we reported no unifying phenotypic and genotypic traits of transmission in 9 HIV-1 transmission pairs suggesting a strong influence of stochastic processes. When re-analyzing envelope (env) sequences of these 9 pairs, Gonzalez et al. concluded that transmission in our cohort is under strong selection for the A55-D57-N88 motif in gp120 and is thus not a stochastic process.

Gonzalez et al. touch in their correspondence on an interesting question that has not been conclusively addressed in the literature: How can we distinguish between virus traits that are linked with transmission and those that generally affect virus fitness?

Dissecting general fitness impairments and effects on transmission is particularly important when dealing with a highly conserved signature like A55-[DK]57-N88. Unlike in the analyses conducted earlier examining SIV, SHIV and HIV-1 transmitter/founder virus sequences, which Gonzalez et al. refer to [3, 4], in our study, the A55-D57-N88 motif is present in the majority of transmitter and recipient viruses (see Figure 1, Gonzalez correspondence). Considering the lack in diversity of this site in our cohort, it is in our opinion difficult to ascertain, and thus potentially misleading, to conclude that the A55-[DK]57-N88 signature is under strong selection during transmission in our cohort. Importantly, as Gonzalez et al. highlight, the A55-[DK]57-N88 signature is highly conserved in chronic HIV-1 infection, suggesting that mutations in this location are associated with a strong fitness cost for the virus. Unfortunately, fitness effects linked with the AKN motif have not yet been experimentally tested, but we completely agree with Gonzalez et al. that this is the most plausible explanation. However, if a site needs to be conserved in chronic infection to ensure fitness it is to be expected that preferentially such virus variants that carry the correct motif succeed in transmission. Indeed, this is what Smith et al. observed when studying SIV transmission using challenge stocks that harbored a considerable fraction of virus variants that lacked this motif [4]. In conclusion all data available on the A55-[DK]57-N88 motif point to a high conservation through all disease stages suggesting an important function for virus fitness. The fact that this site is also preserved in transmission thus needs to be viewed in the context of the general importance of this site rather than a specific trait linked with transmission. Thus, general fitness sustaining sites are expected to be important and required to be conserved throughout all stages of the infection including transmission and thus cannot not serve as ideal genetic traits that distinguish selection during the transmission process from selection during other stages of the infection.

When criticizing our use of the term “stochastic process”, Gonzalez et al. touch on another important issue in the field, namely the lack of a unifying definition of the terms selection and stochastic processes. We used the term stochastic process to describe a process with underlying stochasticity to distinguish this process from a process based on pure selection. Outside of mathematics, the term “stochastic” is very often used in this context. Gonzalez et al. define a “stochastic process” as “a random function” (with the implicit understanding that “random” means “completely random”, see below). In the strict mathematical sense, however, a stochastic process is a set of random variables, normally indexed over a set describing time. When it comes to transmission of an HIV strain, each viral strain that enters a new host with the viral inoculum has a certain probability to start infection. Some strains might have higher probability than others or even 0 probability to start host infection. Thus the correct mathematical description of which viral strain ends up being the founder strain of a new infection would be a single random variable and not a “stochastic process” in the strict mathematic sense. One can distinguish two extreme cases: (1) the transmitted viral strain is chosen uniformly at random from the transmitter’s viral population, i.e. each single viral strain of the transmitter’s viral population has the same probability to start the infection in the recipient and (2) the choice of the transmitted strain happens by pure selection, i.e. only one viral strain will be transmitted with probability 1. In the literature a clear definition of what selection means is missing and ranges somewhere in between these two extreme cases. Note however, that both cases can be modelled as random variables in a mathematically strict sense. We tend to a definition of selection in which viral strains having specific phenotypic characteristics end up being transmitted in each transmission event. This means that the probability distribution over the state space of viral strains is skewed towards strains with a specific phenotype. We did not find evidence for this definition of selection in our cohort of 9 transmission pairs.

Our study aimed to explore phenotypic properties of transmitter and recipient viruses to define whether T/F viruses bear traits that are under selection during transmission. We analyzed several phenotypic characteristics such as neutralization sensitivity, replication capacity, cell–cell versus cell-free virus transmission, IFN sensitivity, and entry kinetics. We additionally investigated genotypic characteristics such as Env variable loop length and glycosylation because these features were already associated with variants more likely to be transmitted in some but not other studies. However, in discussing our findings we stated: “In sum, with the possible exception of increased sensitivity to IFNα, our study revealed no phenotypic feature that was linked with transmission strongly suggesting that transmission is to a large proportion stochastic. Nevertheless, this does not rule out that selective determinants exist.”

Thus, in our view we carefully stated our principle findings and did not over-interpret the lack of finding specific transmission traits. Further, in agreement with Gonzalez et al. we proposed that larger studies with confirmed transmission pairs are crucial to further explore to what extent transmission is governed by stochastic or selective forces. As we pointed out, the analyses of transmission remain complex as the earliest stages of transmission cannot be examined in vivo and findings are therefore based on in vitro phenotypic and genotypic analysis of the closest possible samples to transmission.

In conclusion, we thank Gonzales et al. for their stimulating report on our paper. While we do not agree that their analyses lead to a different interpretation of our data than presented earlier [2], their commentary raised a number of interesting questions and highlights the need for larger phenotypic and genotypic studies to unravel the contribution of selective versus stochastic processes to HIV-1 transmission.

## References

[1] Gonzalez M, DeVico AL, Spouge JL (2017). Conserved signatures indicate HIV-1 transmission is under strong selection and thus is not a “stochastic” process. Retrovirology..

[2] Oberle CS, Joos B, Rusert P, Campbell NK, Beauparlant D, Kuster H, Weber J, Schenkel CD, Scherrer AU, Magnus C (2016). Tracing HIV-1 transmission: envelope traits of HIV-1 transmitter and recipient pairs. Retrovirology.

[3] Gonzalez MW, DeVico AL, Lewis GK, Spouge JL (2015). Conserved molecular signatures in gp120 are associated with the genetic bottleneck during simian immunodeficiency virus (SIV), SIV-human immunodeficiency virus (SHIV), and HIV type 1 (HIV-1) transmission. J Virol.

[4] Smith SA, Kilgore KM, Kasturi SP, Pulendran B, Hunter E, Amara RR, Derdeyn CA (2015). Signatures in Simian immunodeficiency virus SIVsmE660 envelope gp120 are associated with mucosal transmission but not vaccination breakthrough in *Rhesus Macaques*. J Virol.

